# Identification of Loci and Candidate Genes Responsible for Pod Dehiscence in Soybean via Genome-Wide Association Analysis Across Multiple Environments

**DOI:** 10.3389/fpls.2019.00811

**Published:** 2019-06-21

**Authors:** Dezhou Hu, Guizhen Kan, Wei Hu, Yali Li, Derong Hao, Xiao Li, Hui Yang, Zhongyi Yang, Xiaohong He, Fang Huang, Deyue Yu

**Affiliations:** ^1^National Center for Soybean Improvement, National Key Laboratory of Crop Genetics and Germplasm Enhancement, Jiangsu Collaborative Innovation Center for Modern Crop Production, Nanjing Agricultural University, Nanjing, China; ^2^Jiangsu Yanjiang Institute of Agricultural Sciences, Nantong, China; ^3^School of Life Sciences, Guangzhou University, Guangzhou, China

**Keywords:** soybean, pod dehiscence, GWAS, haplotype analysis, *Glyma09g06290*, polymorphism analysis

## Abstract

Pod dehiscence (shattering) is the main cause of serious yield loss during the soybean mechanical harvesting process. A better understanding of the genetic architecture and molecular mechanisms of pod dehiscence is of great significance for soybean breeding. In this study, genome-wide association analysis (GWAS) with NJAU 355K SoySNP array was performed to detect single nucleotide polymorphisms (SNPs) associated with pod dehiscence in an association panel containing 211 accessions across five environments. A total of 163 SNPs were identified as significantly associated with pod dehiscence. Among these markers, 136 SNPs identified on chromosome 16 were located in the known QTL *qPDH1*. One, one, three, eleven, three, one, three, three and one SNPs were distributed on chromosome 1, 4, 6, 8, 9, 11, 17, 18, and 20, respectively. Favorable SNPs and six haplotypes were identified based on ten functional SNPs; among those Hap2 and Hap3 were considered as optimal haplotypes. In addition, based on GWAS results, the candidate gene *Glyma09g06290* was identified. Quantitative real-time PCR (qRT-PCR) results and polymorphism analysis suggested that *Glyma09g06290* might be involved in pod dehiscence. Furthermore, a derived cleaved amplified polymorphic sequences (dCAPS) marker for *Glyma09g06290* was developed. Overall, the loci and genes identified in this study will be helpful in breeding soybean accessions resistant to pod dehiscence.

## Introduction

Soybean [*Glycine max* (L.) Merr.] is one of the major oil crops and provides edible oil and abundant protein for human beings. Soybean pod dehiscence (shattering) is when mature pods open along their dorsal or ventral sutures to release seeds (Kang et al., 2005). As pod dehiscence leads to significant yield loss in soybean and may cause more damage to soybean production as climatic conditions become harsher, breeding for soybean varieties with pod-shattering resistance is always one of the important goals for breeders.

In the past two decades, a few QTLs or genes controlling pod dehiscence have been identified. Previously, QTL analysis in cultivated soybean has revealed a possible major QTL and several minor QTLs that regulate pod dehiscence. Bailey et al. (1997) first identified the major QTL by using 140 restriction fragment length polymorphism (RFLP) markers in a population of 120 F4-derived lines (Bailey et al., 1997). Funatsuki et al. (2006) detected this major QTL and named it *qPDH1*; it was located between the markers Sat_093 and Sat_366 on chromosome 16 and accounted for 50% of the total variance (Funatsuki et al., 2006). Furthermore, this QTL was also reported by several other studies that used different populations and genetic backgrounds (Liu et al., 2007; Kang et al., 2009; Yamada et al., 2009; Suzuki et al., 2010; Ju et al., 2017), suggesting that *qPDH1* may be the major QTL associated with pod dehiscence. In addition, other QTLs that were deemed to be minor were also mapped (Bailey et al., 1997; Liu et al., 2007; Kang et al., 2009; Yamada et al., 2009).

Pod dehiscence/seed shattering has been deeply investigated in many crops (Vittori et al., 2019), several genes has been identified in rice (Konishi et al., 2006; Zhou et al., 2012), common bean (Koinange et al., 1996; Rau et al., 2019), *Medicago* (Fourquin et al., 2013), and cowpea (Suanum et al., 2016; Lo et al., 2018). In soybean, studies predicted that two to four genes were responsible for pod dehiscence (Caviness, 1969; Tsuchiya, 1986, 1987; Kang et al., 2009). To date, two genes located on chromosome 16 have been cloned and characterized. A gene, *Pdh1* (*Glyma16g25580*), detected by fine mapping (located at the *qPDH1* QTL) and encoding a dirigent-like protein, promotes pod dehiscence by increasing the torsion of dried pod walls (Funatsuki et al., 2014). In addition, Dong et al. (2014) identified a *NAC* gene, *SHAT1-5* (*Glyma16g02200*) by analyzing the changes due to domestication between cultivated soybean and wild soybean. This gene activates secondary-cell-wall biosynthesis and promotes the thickening of fiber-cap cells in pod sutures (Dong et al., 2014).

Although linkage mapping has been proved a powerful method to explore regions of the genome that co-segregate with a given trait, it is ordinarily difficult to isolate candidate genes based on a single QTL mapping experiment due to the limited markers (Zhang et al., 2014). Compared to linkage mapping, GWAS can be applied in germplasm collections or naturally occurring populations and provides higher resolution in terms of defining the genomic positions of genes or QTLs (Shirasawa et al., 2013). In recent years, with the advance of next-generation sequencing (NGS), this approach has been successfully applied to important traits in many plant species, including *Arabidopsis thaliana* (Kobayashi et al., 2016; Kalladan et al., 2017), rice (Huang et al., 2010; Yano et al., 2016) and maize (Kump et al., 2011; Wang X. et al., 2016). In soybean, GWAS has been used to explore markers associated with many traits. For example, Hao et al. (2012) used GWAS to identify 19 SNPs and five haplotypes for soybean yield and yield components (Hao et al., 2012); Chu et al. (2017) mapped the *GmMYB29* (*Glyma20g35180*) gene related to isoflavone biosynthesis in soybean via GWAS (Chu et al., 2017); and Zhang et al. (2018) identified 87 chromosomal regions for seed composition based on 31,850 SNPs by GWAS (Zhang et al., 2018).

Although previous studies have identified many QTLs controlling pod dehiscence in soybean, these QTLs were detected using classical linkage analysis with a limited number of markers, and the allelic variation was also limited by using RILs or other linkage mapping populations. Furthermore, to date, only two pod-dehiscence-related genes have been cloned. In addition, we know little about the other QTLs related to pod dehiscence.

In our previous work, we performed primary mapping of pod-dehiscence-related QTLs due to the low density of the genetic map (Hu, 2017). In this paper, to obtain more accurate information regarding pod-dehiscence-related SNPs, GWAS with the large genome-wide NJAU 355K SoySNP array was performed across multiple environments. The aims of our study were as follows: (i) identify loci and candidate genes involved in pod dehiscence; (ii) identify favorable haplotypes relevant to pod dehiscence. These results will be helpful for MAS in soybean breeding programs.

## Materials and Methods

### Materials and Field Trials

In this study, the association panel containing 211 soybean accessions was used (including 183 landraces, 25 improved accessions and three accessions with unknown evolution type). This population was collected from different geographic origins that exhibit variant phenotypes for pod dehiscence, and provided by the National Center for Soybean Improvement of China. Experiments to assess association panel were sowed in two places from 2013 to 2017: Jiangpu Experimental Station (32°12 N 118°37 48 E) in 2013 and 2014 (designated as environment Env1 and Env2, respectively); and the Experimental Farm of Jiangsu Yanjiang Institute of Agricultural Sciences (31°58 48 N 120°53 24 E), Nantong, China, in 2015–2017 (designated as Env3, Env4, and Env5, respectively). A complete randomized block design with three replications was used for all trials. Each line of the populations was planted in three rows, and each row was 200 cm long with 50 cm row spacing. At the seedling stage (about 2 weeks after germination), the number of plants in per row was limited to 15–20. Nutrition, water supply, weeding and insect control were maintained throughout the experiment.

### Evaluation of Pod Dehiscence

Three plants per line were harvested randomly, and twenty pods per plant were examined. The maturity pods were stored in a kraft paper bag after harvested and then put in the heating ovens as soon as possible. The degree of pod dehiscence was identified by monitoring the percentage of dehiscent pods after heat treatment at 60°C for 3 h (Funatsuki et al., 2006; Yamada et al., 2009). As shown in Supplementary Figure S1, after heat treatment, the completely opened pods were used to calculate PPD. PPD = (number of pod dehiscence/total pods) × 100% (Yamada et al., 2009). The germplasm number, name, origin and phenotypic data of the association panel are listed in Supplementary Table S1.

### Phenotypic Data Analysis

The phenotypic data of the association panel was determined using the mean values for each line (Env1 to Env5) with the R software (R Development Core Team, 2015), including descriptive statistics, ANOVA, and *h*^2^. Variable components (genotype, year, line, and location) were evaluated in R with the “lme4” package using the BLUP model. The BLUP model was based on the following formula (Merk et al., 2012):

$$Y_{i\,k}=\mu +G_{i}+Y_{k}+G\,Y_{i\,k}+\varepsilon_{i\,k}$$

In this formula, $Y_{i\,k}$ is the trait studied, μ is the overall mean, $G_{i}$ is the ith genotypic effect, $Y_{k}$ is the effect of the kth year, $G\,Y_{i\,k}$ is the interaction of genotype × year, and $\varepsilon_{i\,k}$ is the residual error. The BLUP was also included as an environment in the GWAS analysis. The $h^{2}$ of pod dehiscence was calculated using the following formula:

$$h^{2}=\sigma_{g}^{2}/\left(\sigma_{g}^{2}+\sigma_{g\,e}^{2}/n+\sigma_{e}^{2}/n\,r\right)$$

In which $h^{2}$, broad-sense heritability; $\sigma_{g}^{2}$, genotype variance; $\sigma_{g\,e}^{2}$, interaction variance of genotype × environment; $\sigma_{e}^{2}$, variance of error components; n, the number of environments; and r, the number of replications.

### Genome-Wide Association Analysis

The 211 soybean accessions were genotyped with the NJAU 355K SoySNP array containing 282,469 SNPs (Wang J. et al., 2016). After filtering out SNPs with MAFs ≤ 0.05, a total of 201,915 SNPs remained. GWAS was conducted based on the MLM model (Q + K) using the TASSEL software V5.0 (Bradbury et al., 2007). The kinship matrix (K) and population structure (Q) were calculated by TASSEL software V5.0 and STRUCTURE software version 2.3.4, respectively (Pritchard et al., 2000; Falush et al., 2003). A FDR of 0.05 was used as a threshold for significant association (Benjamini and Hochberg, 1995). Manhattan plots and Q–Q plots were generated using the R software package “qqman” (Turner, 2014).

### Digital Expression Data Analysis

Two databases were used to identify the tissue expression patterns of candidate genes. RNA-Seq data from 14 tissues, including three vegetative tissues (leaves, root, and nodules) and the whole seeds from 11 stages of reproductive tissue development (flower, pod, and seed), were downloaded from SoyBase^1^, and soybean microarray expression data was downloaded from Plant Expression Database^2^ (GEO Accession: GSE26443). The heat maps were generated by using R software packages “heatmap.”

### Candidate Gene Selection and Gene Expression Analysis

Based on the LD decay distance of significant SNPs in the GWAS results, we predicted the putative genes related to pod dehiscence according to the soybean genome annotation (Wm82.a1.v1.1^3^). Meanwhile, BLASTP analysis was also performed against *Arabidopsis* proteins using the amino acid sequences of these putative genes.

The soybean accession Williams 82 was selected to analyze the tissue expression patterns of the putative genes. First, seeds were grown in growth chambers under the conditions of 16/8 h (day/night), 28/25°C (day/night), and 60% relative humidity. Then, roots, stems, leaves, and flowers were sampled during the full-bloom period; pods were sampled on the 7th, 15th, 25th, and 45th days after flowering (DAF), and mature seeds were also collected. The expression levels of the putative genes were detected in 15-day pods in a subset of 18 soybean accessions, representing varieties with high and low PPD. Total RNA was isolated using a Plant RNA Extract Kit (TianGen, Beijing, China), and cDNA was synthesized using a TaKaRa Prime Script^TM^ RT reagent kit according to the manufacturer’s instructions. Gene expression in different tissues was performed by qRT-PCR assays using an ABI 7500 system (Applied Biosystems, Foster City, CA, United States) with SYBR Green Real-time Master Mix (Toyobo). Three biological and three technical replicates were used. The constitutively expressed soybean tubulin gene (GenBank accession number: AY907703) was used as a control. The normalized expression, reported as fold change, was calculated for each sample as $\Delta \,\Delta \,C\,T={\left(C\,T_{T\,a\,r\,g\,e\,t}-C\,T_{T\,u\,b\,u\,l\,i\,n}\right)}_{g\,e\,n\,o\,t\,y\,p\,e}-{\left(C\,T_{T\,a\,r\,g\,e\,t}-C\,T_{T\,u\,b\,u\,l\,i\,n}\right)}_{c\,a\,l\,i\,b\,r\,a\,t\,o\,r}$ (Livak and Schmittgen, 2001). The primers used are listed in Supplementary Table S2.

### Gene Structure Analysis

GSDS^4^ was used to analyze the gene structure. The protein sequences of homologous genes from different plants were obtained from NCBI database^5^ (accession numbers are listed in Supplementary Table S3). Sequence alignment was analyzed using ClustalX software version 1.83 (Jeanmougin et al., 1998). A NJ phylogenetic tree was constructed based on protein sequences with MEGA 6.0 software using bootstrap method with 1,000 replications.

### Haplotype Analysis

A pair of gene-specific primers was designed (Primer 5.0) to amplify *Glyma09g06290* from 42 soybean accessions (forward: 5′-CCAAACAGAGTGAGTGACTTG-3′, reverse: 5′-TGCTACTTTCTTCTTCTAGC-3′). These sequences were aligned using the ClustalX software version 1.8, and the SNPs with MAFs ≥ 0.05 were identified among these accessions. The LD level was calculated by the HapView 4.0 software program (Barrett et al., 2005). The correlation between the expression of *Glyma09g06290* and pod dehiscence was calculated by SPSS 20.0 software (Pearson correlation). In addition, the haplotype block was defined by the “Solid Spine of LD” algorithm. LD analysis of 112 significant SNPs located on chromosome 16 was performed using the R software package “LDheatmap” (Shin et al., 2006).

### dCAPS Marker Development

The dCAPS marker of *Glyma09g06290* was developed based on the S_-500 (C/G). The gene-specific primers were designed by dCAPS Finder 2.0^6^ (forward: 5′-CTCATATCTTT GTGTTGTCCATG-3′, reverse: 5′-ATTGGAGTGATAAGGAAA CTGG-3′). The PCR products were digested with restriction endonuclease *Nco*I (37°C, 1 h). Digestion products were assayed by 8% polyacrylamide gel electrophoresis to distinguish the polymorphic fragments.

## Results

### Phenotypic Variation in Association Panel

**FIGURE 1:**
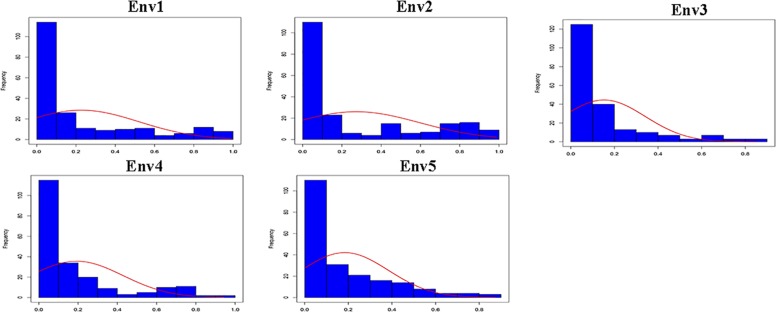
Frequency distribution of the PPD in association panel for five environments (Env1, Env2, Env3, Env4, and Env5).

Descriptive statistics, ANOVA and *h*^2^ of the PPD were identified for the association panel in five environments (Table 1). The PPD for the individual accessions in the association panel ranged from 0 to 0.99, and the average value was between 0.15 and 0.27. *h*^2^ was estimated as 78.5% in association panel, which was slightly lower than that found in RILs (Kang et al., 2005). The frequency distribution showed a skewed distribution (Figure 1), not a normal distribution, which is similar to the previously reported results (Kang et al., 2009). In addition, the ANOVA results indicated that pod dehiscence was significantly influenced by the genotype effect and the interaction effects of genotype and environment (*P* < 0.001) in association panel.

**TABLE 1:** Descriptive statistics, ANOVA and broad-sense heritability of the PPD in five environments of association panel (Env1, Env2, Env3, Env4, and Env5).

**Env**	**Mean**	**SD**	**Median**	**Minimum**	**Maximum**	**CV**	**Skewness**	**Kurtosis**	**G**	**G × E**	***h*^2^**
Env1	0.23	0.30	0.08	0	0.99	130.4%	1.24	0.17	^∗∗∗^	^∗∗∗^	78.5%
Env2	0.27	0.32	0.09	0	0.99	118.5%	0.91	–0.77	^∗∗∗^	^∗∗∗^	
Env3	0.15	0.19	0.08	0	0.86	126.6%	1.91	3.03	^∗∗∗^	^∗∗∗^	
Env4	0.19	0.24	0.09	0	0.94	126.3%	1.59	1.35	^∗∗∗^	^∗∗∗^	
Env5	0.20	0.23	0.09	0	0.96	115.0%	1.44	1.07	^∗∗∗^	^∗∗∗^	

*Env, environment; SD, standard deviation; CV, coefficient of variation; G, genotype; G × E, genotype × environment; *h^2^*, broad-sense heritability. ^∗∗∗^Significant at *P* < 0.001.*

### Genome-Wide Association Analysis for Pod Dehiscence

QTL mapping suffers from fundamental limitations; one of these is the limited allelic diversity between recombinant inbred population and parents (Korte and Farlow, 2013). In contrast, by taking advantages of the abundant natural variations of a population, GWAS is helpful for favored allele detecting and functional SNP discovery. In this study, to mine the SNPs significantly associated with pod dehiscence, GWAS was conducted using 201,915 SNPs (NJAU 355K SoySNP array) with the MLM (Q + K) model in an association panel including 211 accessions.

**FIGURE 2:**
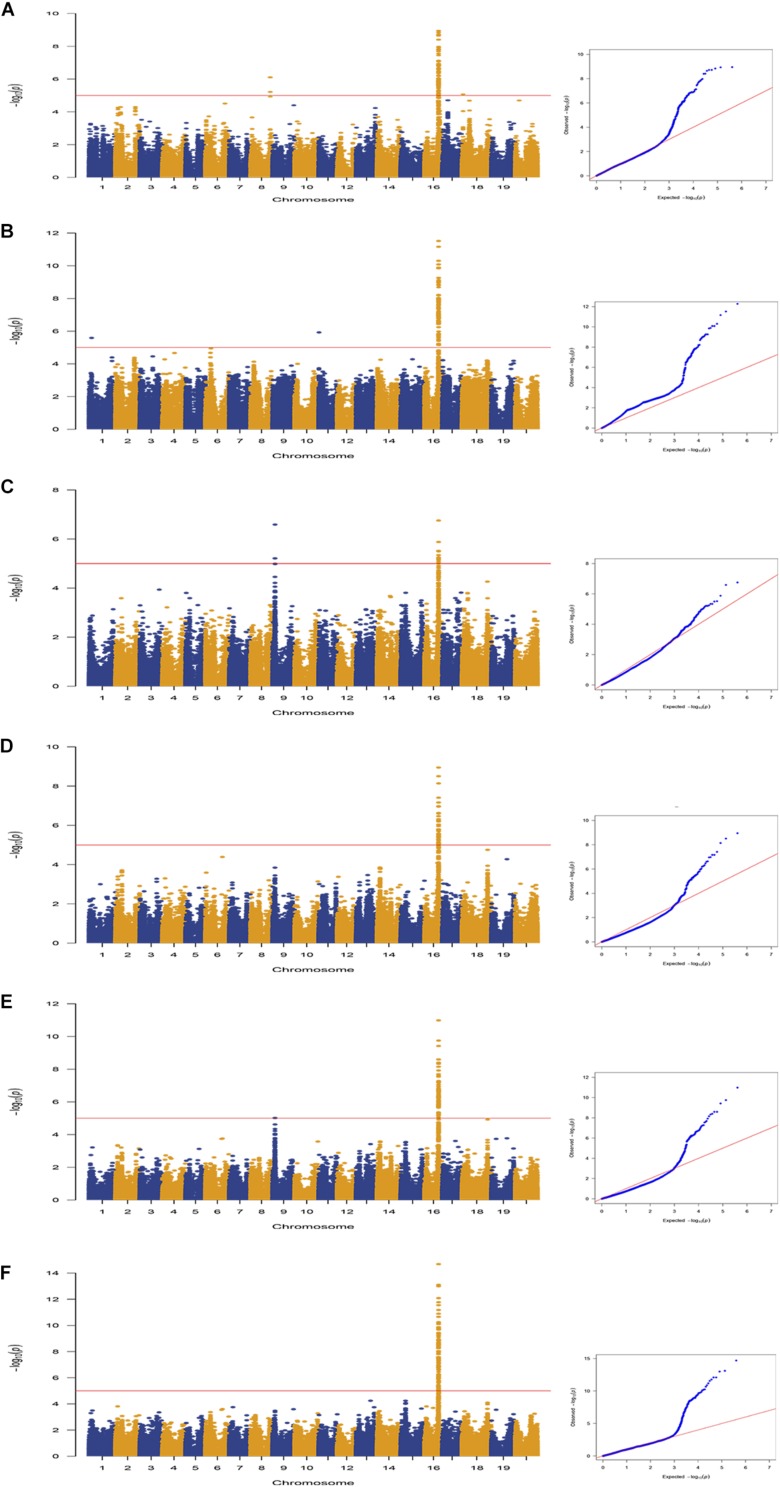
Genome-wide association study of pod dehiscence in association panel using the MLM (Q + K) model across multiple environments. Manhattan plots and Q–Q plots for pod dehiscence in the following environments: **(A)** Env1; **(B)** Env2; **(C)** Env3; **(D)** Env4; **(E)** Env5; and **(F)** BLUP. The horizontal line depicts the significance threshold when FDR = 0.05.

A total of 163 SNPs were identified as significantly associated with pod dehiscence in five environments and BLUP (Figure 2, Table 2, and Supplementary Table S4). Among these SNPs, 136 SNPs were located on chromosome 16 in multiple environments. Among these 136 SNPs, one SNPs was detected in five environments and BLUP. In addition, 51 SNPs were identified in four environments and BLUP; 18 SNPs were mapped in three environments and BLUP; and 19 SNPs were mapped in two environments and BLUP. These 136 SNPs were clustered on chromosome 16 from 29135922 to 29865027 (∼729 kb), which completely overlapped with two QTLs (*Pod dehiscence 2-1* and *Pod dehiscence 3-4*) and partially overlapped with *Pod dehiscence 1-7* and *Pod dehiscence 4-2* (Bailey et al., 1997; Funatsuki et al., 2006; Kang et al., 2009; Yamada et al., 2009). The gene *pdh1*, controlling pod dehiscence in soybean, was also located in this cluster. In addition, a few significant SNPs were distributed on other chromosomes, such as chromosome 1, 4, 6, 8, 9, 11, 17, 18, and 20 (Figure 2, Table 2, and Supplementary Table S4). On chromosome 9, two SNPs were identified in both Env3 and Env5 as significant SNPs.

**TABLE 2:** SNP loci that significantly associated with pod dehiscence based on GWAS in the association panel.

**Chr**	**MSS**	**MSS Position**	**Mss *P*-value**	**Significant region**		**No. of SNPs**	**Env**	**Related QTL or gene identified in previous studies**
							
				**Start**	**End**			
1	AX-93963018	5388850	2.58 × 10^–06^	5258850	5518850	1	Env2	–
4	AX-93708700	25726440	2.17 × 10^–05^	25596440	25856440	1	Env2	–
6	AX-93727683	11784406	1.13 × 10^–5^	10563310	41546032	3	Env2 (2), Env1 (1)	–
8	AX-93930129	42050788	7.83 × 10^–07^	41864756	42186397	11	Env1	*Pod dehiscence 4-1* (Yamada et al., 2009)
9	AX-93762848	5145974	2.58 × 10^–07^	4940686	5275974	3	Env3 Env5 (2), Env3 (1)	–
11	AX-94082716	577915	1.19 × 10^–06^	447915	707915	1	Env2	
16	AX-93853895	29601807	2.06 × 10^–15^	29365082	29736722	1	Env1 Env2 Env3 Env4 Env5 BLUP	
16	AX-93853825	29421300	5.78 × 10^–10^	29194421	29850274	51	Env1 Env2 Env4 Env5 BLUP	*Pod dehiscence 1-7* (Bailey et al., 1997) *Pod dehiscence 2-1* (Funatsuki et al., 2006) *Pod dehiscence 3-4* (Kang et al., 2009) *Pod dehiscence 4-2* (Yamada et al., 2009) *Pod wall width 1-9* (Guang-yu et al., 2011) *pdh1* (Funatsuki et al., 2014)
16	AX-93853894	29600010	1.29 × 10^–10^	29136226	29870913	18	Env1 Env2 Env5 BLUP (14) Env1 Env4 Env5 BLUP (4)	
16	AX-93853796	29340570	2.84 × 10^–08^	29210570	29879081	19	Env1 Env2 BLUP (16) Env2 Env5 BLUP (3)	
16	AX-93946746	29215338	1.15 × 10^–07^	29005922	29968491	11	Env1 BLUP	
16	AX-93853865	29545423	5.30 × 10^–07^	29018509	29990917	36	Env1 (12), Env2 (4), BLUP (20)	
17	AX-94289430	11868679	1.98 × 10^–5^	11738679	12008912	3	Env1	–
18	AX-94165965	2292799	8.60 × 10^–06^	2162799	54328119	3	Env1	–
20	AX-93901310	8202869	2.03 × 10^–05^	8072869	8332869	1	Env1	–

*Chr, chromosome; MSS, most significant SNP; Significant region, significant region was defined as the LD decay distance flanking the significant SNPs; No. of SNPs, number of significant SNPs; Env, environment. The number in brackets indicates the number of significant SNPs detected in different environments.*

### Identification of Favorable SNPs and Haplotypes Relevant to Pod Dehiscence

**FIGURE 3:**
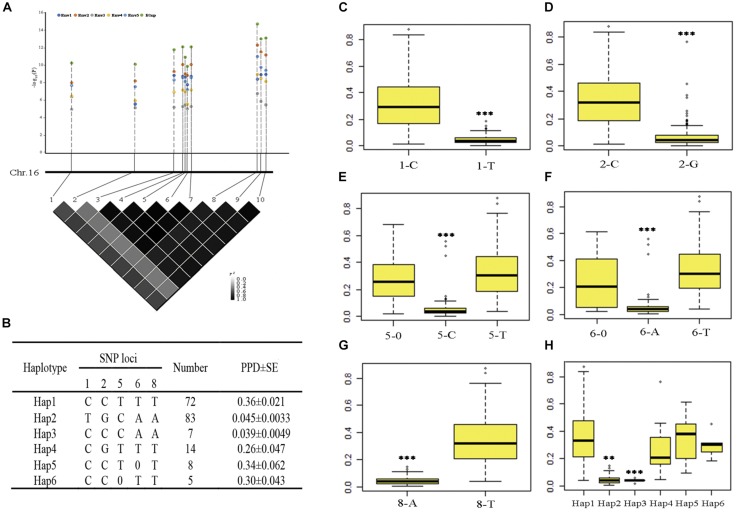
Haplotype analysis of ten SNPs significantly associated with pod dehiscence in five environments and the PPD phenotype of five significant SNPs. **(A)** The physical locations of 10 SNPs and the LD plot based on pairwise *R*^2^-values between these SNPs. The color bar was used to indicate the *R*^2^-values. **(B)** Haplotypes observed in the association panel using the 10 SNPs; 0 indicates a base deletion. **(C–G)** The PPD phenotype of five significant SNPs. **(H)** Comparison of PPD among the haplotypes Hap1, Hap2, Hap3, Hap5, and Hap6. ^∗∗∗^Significant at *P* < 0.001. Two tail *t*-test was used for statistical analysis. 1–10 represent the SNPs AX-93853844, AX-94151086, AX-93853870, AX-93853873, AX-93853874, AX-94151101, AX-93853876, AX-93853895, AX-93853896, and AX-94151124; SE, standard error.

One SNP was mapped in both five environments and BLUP on chromosome 16; we also found that nine SNPs were mapped in four environments and BLUP on chromosome 16 (the *P*-value of nine SNPs in Env3 was as low as 9.91 × 10^–06^); the *R*^2^ ranged from 11.9 to 38.5% (Table 2 and Supplementary Table S4). These significant SNPs have more allelic variations and can represent the quality of pod dehiscence in our association panel. Therefore, these SNPs were used to analyze the haplotypes related to pod dehiscence in soybean. We selected 189 of 211 accessions from different geographic origins for further haplotype analysis. Ten significant loci showed very strong LD level and formed one block. The number 1–10 represents these ten SNPs AX-93853844, AX-94151086, AX-93853870, AX-93853873, AX-93853874, AX-94151101, AX-93853876, AX-93853895, AX-93853896, and AX-94151124, respectively (Figure 3A and Supplementary Table S5). Of which, the SNP 8 and 3, 4, 7, 9, 10 showed complete LD. When 8 is T base, 3, 4, 7, 9, 10 is C, T, G, A, T base, respectively; when 8 is A base, 3, 4, 7, 9, 10 is A, C, A, T, C base, respectively (Figure 3B). Therefore, these ten SNPs were subdivided into five SNPs (1, 2, 5, 6, and 8). Based on the five SNPs within the LD block, six haplotype classes were observed in the 189 accessions (Hap1-Hap6) (Figure 3B). Hap2 (TGCAA) was the largest group (*n* = 83); and Hap1 (CCTTT) was the second largest group (*n* = 72). The other haplotypes, Hap3 (CCCAA), Hap4 (CGTTT), Hap5 (CCT0T), and Hap6 (CC0TT), comprised seven, fourteen, eight and five accessions, respectively.

The phenotypic data of five significant SNPs and six haplotypes were further analyzed. In total, 117 accessions with 1-C exhibited significantly higher values than 1-T; 104 accessions with 2-C exhibited significantly higher values than 2-G; 101 accessions with 5-T and 12 accessions with 5-0 exhibited significantly higher values than 5-C; 98 accessions with 6-T and 14 accessions with 6-0 exhibited significantly higher values than 6-A, 109 accessions with 8-T exhibited significantly higher values than 8-A (Figures 3C–G). In addition, Hap1 and Hap5 had significantly higher PPD than Hap2 and Hap3 (Figure 3H). Considering that soybean varieties with pod-shattering resistance are one of the major goals for breeders, 1-T, 2-G, 5-C, 6-A, and 8-A were the favorable markers; Hap2 and Hap3 should be the favorable haplotypes.

Hap1 and Hap2 were not only the unfavorable and favorable haplotypes but also the largest and second largest groups, respectively. The number of Hap3 and Hap5 was limited (seven and eight accessions, respectively). Therefore, Hap1 and Hap2 were used to analyze the geographical distribution of soybean landraces with different pod dehiscence traits. After filtering out accessions with indeterminate ecoregions, 66 and 76 accessions remained in Hap1 and Hap2, respectively. Hap1 was observed in the three main growing ecoregions in China (Tian et al., 2010) and was mainly (75.8%) distributed in the southern ecoregion (SR); Hap2-containing accessions were mostly observed in the Huang-Huai ecoregion (HR) (34.2%) and SR (34.2%) (Table 3). These results revealed that the soybean landraces of Chinese origin possessing the elite haplotype were mostly distributed in HR and SR.

**TABLE 3:** Geographic distribution of Hap1 and Hap2 in three soybean-growing ecoregions (NR, HR, and SR) in China.

**Haplotype**	**Number**				
	
	**NR**	**HR**	**SR**	**HR/SR**	**NR/HR**
Hap1	3 (4.6%)	2 (3.0%)	50 (75.8%)	9 (13.6%)	2 (3.0%)
Hap2	9 (11.8%)	26 (34.2%)	26 (34.2%)	8 (10.5%)	7 (9.3%)

*NR, northern ecoregion; HR, Huang-Huai ecoregion; SR, southern ecoregion.*

### Identification of Putative Genes

In GWAS results, under the threshold of FDR < 0.05, we detected three significant SNPs on chromosome 9. Moreover, two of them were close to each other and repeatedly identified in different environments. Interestingly, in other regions, there was no significant SNP that was repeatedly detected except on chromosome 16. Notably, one of these three SNPs, AX-93762848, was also detected by using MLMM model (Supplementary Figure S2). It demonstrates that these three SNPs on chromosome 9 were essential to dehiscence trait in soybean. Therefore, the candidate genes were selected based on the SNPs on chromosome 9.

There were 26 genes detected in the 130 kb flanking regions (LD decay distance of cultivated soybeans) of the two SNPs (Table 4; Wang J. et al., 2016). We then performed a BLASTP analysis using the *Arabidopsis* genome against these 26 genes. The results showed that two genes were without any explicit biological function annotations. Among the other 24 genes with functional annotations, *Glyma09g06320* and *Glyma09g06390* were predicted to be involved in cellular differentiation and cell wall formation. Additionally, the gene *Glyma09g06290* is homologous to *At2g18969* which belongs to *bHLH* (basic helix-loop-helix) gene family. In *Arabidopsis*, two bHLH transcriptional factors (IND and ALC) were reported to be related to silique dehiscence (Rajani and Sundaresan, 2001; Liljegren et al., 2004). The other genes were forecasted to participate in abiotic and biotic stress, transport, metabolic process, and other functions. We also listed the detail information about the orthologs of these genes in *Medicago truncatula*, *Oryza sativa*, and *Zea mays*, respectively (Supplementary Table S6). Moreover, we identified the expression patterns of these 24 genes in the RNA-Seq and microarray data, respectively (Supplementary Figure S3). The RNA-Seq data showed that *Glyma09g06290* was predominately expressed in pod shell and *Glyma09g06390* was expressed in several tissues. However, *Glyma09g06320* was not expressed according to the RNA-seq data, and this result was confirmed by our qRT-PCR result. In the microarray data, we found that *Glyma09g06290* was highly expressed in pod elongation stage and sharply decreased in late seed growth period. By combining the above results, these two genes (*Glyma09g06390* and *Glyma09g06290*) may be related to pod dehiscence.

**TABLE 4:** Genes detected in the 130 kb flanking region of the significant SNPs on chromosome 9.

**Gene ID**	**Homolog**	**Functional annotation in the Phytozome database**
Glyma09g06180	AT5G24080	Protein kinase superfamily protein
Glyma09g06190	AT5G24080	Protein kinase superfamily protein
Glyma09g06200	AT5G24080	Protein kinase superfamily protein
Glyma09g06220	AT5G57360	Clock-associated PAS protein ZTL
Glyma09g06230	AT2G18940	Tetratricopeptide-repeat-like superfamily protein
Glyma09g06250	AT4G30190	P-type ATPase superfamily
Glyma09g06260	AT5G41750	Disease resistance protein
Glyma09g06275	AT5G51630	Disease resistance protein
Glyma09g06290	AT2G18969	bHLH family transcription factor
Glyma09g06300	AT4G25910	NFU domain protein
Glyma09g06310	AT5G57340	Ras guanine nucleotide exchange factor Q-like protein
Glyma09g06320	AT3G62360	Carbohydrate-binding-like fold
Glyma09g06330	AT5G41540	Disease resistance protein
Glyma09g06350	AT2G18980	Peroxidase superfamily protein
Glyma09g06365	AT1G62520	Sulfated surface-like glycoprotein
Glyma09g06380	AT5G57330	Galactose mutarotase-like superfamily protein
Glyma09g06390	AT4G30160	Major actin filament bundling protein
Glyma09g06410	AT1G71840	WD-40 repeat family protein
Glyma09g06431	/	/
Glyma09g06450	AT1G17690	U3 small nucleolar RNA-associated protein
Glyma09g06460	AT3G20250	Arabidopsis Pumilio (APUM) protein
Glyma09g06470	AT2G19080	Metaxin-like protein
Glyma09g06480	AT2G19090	DUF630 family protein
Glyma09g06491	ATCG00905	/
Glyma09g06500	/	Chloroplast gene encoding ribosomal protein s12
Glyma09g06521	AT5G54780	Gyp1p superfamily protein

### Expression Patterns of Putative Genes

**FIGURE 4:**
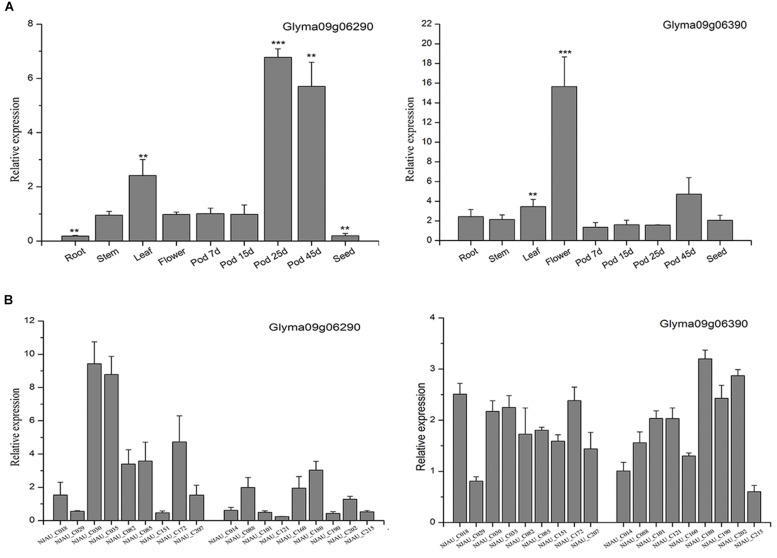
Expression patterns of *Glyma09g06290* and *Glyma09g06390* by quantitative real-time polymerase chain reaction. **(A)** The expression of the putative genes in different tissues; the expression in 7-day pods was used as a control (expression value = 1). **(B)** The expression in 15-day pods in varieties with high and low PPD. Error bar indicates the standard deviation. ^∗∗^Significant at 0.001 < *P* < 0.01, ^∗∗∗^significant at *P* < 0.001. Two tail *t*-test was used for statistical analysis.

According to the Soybase^7^, we listed the candidate genes’ coding product and metabolic processes (Supplementary Table S7). Moreover, to elucidate the potential functions of the two genes, qRT-PCR was performed to study the expression patterns of these two genes. First, the soybean accession Williams 82 was selected to analyze the tissue expression pattern of the putative genes. *Glyma09g06290* was highly expressed in the pod, and the expression of *Glyma09g06290* was significantly increased in the late pod growth period, however, the expression of *Glyma09g06390* was very low in pod growth period (Figure 4A). These results were similar to the public RNA-Seq data (Supplementary Figure S3). Second, 18 soybean accessions representing varieties with high and low PPD were used to analyze the expression patterns in extreme varieties. The results showed that the expression of *Glyma09g06290* rather than that of *Glyma09g06390* in the high-PPD varieties was significantly higher than that in the low-PPD varieties in 15-day pods (Figure 4B). Therefore, *Glyma09g06290* may be involved in pod dehiscence in soybean.

### Sequence Analysis of *Glyma09g06290*

**FIGURE 5:**
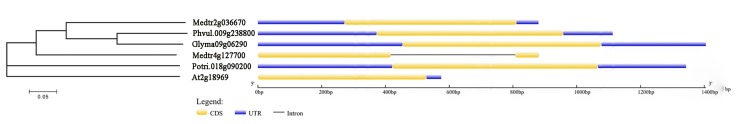
Phylogenetic and gene structure analysis of *Glyma09g06290* and other plant gene. A NJ phylogenetic tree was constructed using full protein sequences. Numbers below branches indicate bootstrap value for 1,000 replicates; CDS, coding sequence; UTR, untranslated transcribed region.

The full genomic length of *Glyma09g06290* is 1405 bp and only contains one exon with 624 bp. Glyma09g06290 protein is composed of 207 amino acids with molecular mass of 23.23 kDa and isoelectric point of 5.39. Further, phylogenetic trees showed that Glyma09g06290 is homologous to *Phaseolus vulgaris* Phvul.009g238800 (identity of 78.7%), *Medicago truncatula* Medtr2g036670 (identity of 61.8%), *Populus trichocarpa* Potri.018g090200 (identity of 61.8%), *M. truncatula* Medtr4g127700 (identity of 50.7%) and *A. thaliana columbia* At2g18969 (identity of 44.9%; Figure 5). By comparing the gene structure of *Glyma09g06290* and other plant homologous genes, we identified that they have only one exon and without intron except *Medtr4g127700* (Figure 5).

### Polymorphisms in the *Glyma09g06290* Gene Are Associated With Pod Dehiscence

**FIGURE 6:**
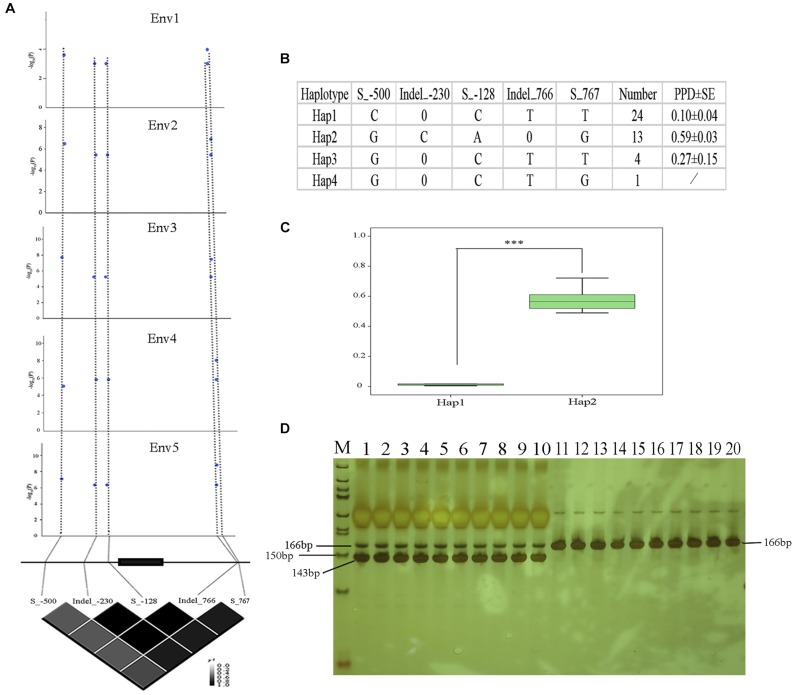
Polymorphisms in *Glyma09g06290* are significantly associated with pod dehiscence. **(A)** *Glyma09g06290*-based association mapping and pairwise LD analysis. Blue dots represent significant variants (Tassel 5.0, GLM model, *P* < 0.01). **(B)** Haplotypes of *Glyma09g06290* among 42 soybean accessions; 0 indicates a base deletion. **(C)** Comparison of PPD between haplotypes Hap1 and Hap2. **(D)** Products of digestion by gel electrophoresis; M: marker, 50 bp DNA ladder (Tiangen, Beijing, China); 1–10: high-PPD varieties NJAU_C008, NJAU_C014, NJAU_C088, NJAU_C101, NJAU_C121, NJAU_C137, NJAU_C160, NJAU_C180 NJAU_C181, and NJAU_C190. Digested products with size of 166 and 143 bp, 23 bp was not observed because of its small molecular weight; 11–20: low-PPD varieties, NJAU_C054, NJAU_C076, NJAU_C080, NJAU_C082, NJAU_C085, NJAU_C098, NJAU_C165, NJAU_C172, NJAU_C201, and NJAU_C216. Undigested product with size of 166 bp. ^∗∗∗^Significant at *P* < 0.001. Two tail *t*-test was used for statistical analysis. SE, standard error.

In order to analyze the association between the allelic variation of *Glyma09g06290* and pod dehiscence, we sequenced the *Glyma09g06290* gene in a subset of 20 accessions with high PPD and 22 accessions with low PPD. An approximately 1.5-kb genomic region, spanning the 5′- to 3′-UTR of *Glyma09g06290*, was analyzed. A total of three SNPs and two indels (insertions and deletions) were identified, including Site_-500 (located 500 bp upstream from the translation start codon, S_-500), Indel_-230, S_-128 in the 5′-UTR, Indel_766 and S_767 in the 3′-UTR (Figure 6). The association study showed that 5 probable causative sites were significantly associated with variations in pod dehiscence (Figure 6A). Furthermore, based on the five significant variants with strong LD, the 42 soybean genotypes were classified into four haplotype classes (Hap1-Hap4). Hap 1 (*n* = 24) is the largest group, and Hap2 (*n* = 13) is the second largest group. Statistically, Hap2 had significantly higher PPD than Hap1 (Figures 6B,C). We then measured the expression of *Glyma09g06290* in pods from 13 of 42 soybean accessions. The expression of this gene was positively correlated with pod dehiscence (*r*^2^ = 0.58, *P* < 0.05) (Supplementary Table S8). Therefore, these results suggested that the expression of *Glyma09g06290* could partially explain the phenotypic variation in pod dehiscence.

### Development of a Functional Marker for *Glyma09g06290*

In this study, five significant SNPs of *Glyma09g06290* were identified and associated with pod dehiscence (Figures 6A–C and Supplementary Figure S4). The S_-500-C exhibited significantly lower values than S_-500-G; Indel_-230-0 exhibited significantly lower values than Indel_-230-C; S_-128-C exhibited significantly lower values than S_-128-A; Indel_766-T exhibited significantly lower values than Indel_766-0; S_767-T exhibited significantly lower values than S_767-G (Figure 6B). Therefore, we developed the dCAPS marker based on one of these SNPs (S_-500). As shown in Figure 6D, we selected ten accessions with high PPD and ten accessions with low PPD. Ten accessions with high PPD produced a 166 and 143 bp amplicons by restriction enzyme digestion. The remaining ten accessions with low PPD produced 166 bp amplicons by restriction enzyme digestion. These results confirmed that dCAPS based on the S_-500 is a functional marker and could be useful for plant breeders.

## Discussion

### Novel Loci Related to Pod Dehiscence Were Identified

The dehiscence of pods (shattering) prior to harvest is a major cause of yield loss in soybean production. Thus, it is extremely important to identify QTLs or genes related to pod dehiscence and apply them to MAS. In this study, by using the association panel which possessed abundant natural variations, GWAS was performed to genotype 211 cultivated soybean accessions with high-quality SNP markers. We identified 163 SNPs. Of which, 136 significant SNPs on chromosome 16 ranged from 29135922 to 29865027 (∼729 kb). These results were consistent with those of a previous study, which showed that the major QTL was located on chromosome 16 (Yamada et al., 2009). Similarly, a very large GWAS also confirmed that shattering in soybean is mainly due to genes located on chromosome 16 (Zhou et al., 2015). Additionally, LD analysis exhibited tight linkage among the 136 associated SNPs (Supplementary Figure S5). One of 136 SNP (AX-93853895) with the lowest *P*-value and maximum *R*^2^ was in the intron of *pdh1*. Consequently, the formation of this SNP cluster on chromosome 16 may be due to the strong effect of *pdh1*. Moreover, the domestication gene related to pod dehiscence between cultivated and wild soybean, *SHAT1-5*, was not mapped in our GWAS results. Thus, the gene effect of *SHAT1-5* is likely to be weak in cultivated soybean accessions.

In addition, we also detected 25 significant SNPs except on chromosome 16 (Table 2). For example, on chromosome 8, one region contains 11 significant SNPs that were significantly associated with pod dehiscence, and this region is close to *Pod dehiscence 4-1*; On chromosome 9, 2 SNPs were co-identified in Env3 and Env5; there are one, one, three, one, three, three and one significant SNPs identified on chromosome 1, 4, 6, 11, 17, 18, and 20, respectively. These SNPs or regions may be the novel loci related to pod dehiscence. Overall, the SNPs identified in this study are helpful to further understanding the genetic basis of soybean pod dehiscence.

### Identification of Favorable Haplotypes Related to Pod Dehiscence in Chinese Soybean Germplasms

Haplotype analysis of a candidate gene or peak SNPs have been reported for soybean (Zhang et al., 2014), rice (Famoso et al., 2011), and cotton (Su et al., 2018). For example, in cotton, four peak SNPs located on chromosome D03 were simultaneously associated with four plant architecture component traits. The four peak SNPs revealed five haplotypes, and Hap2 was the most favorable haplotype. In our study, ten significant SNPs were associated with pod dehiscence in Env1-Env5 and BLUP. Haplotype analysis showed that the 10 SNPs could be found as six haplotypes (Hap1-Hap6). Hap2 and Hap3 had lower PPD than the other haplotypes, demonstrating that Hap2 and Hap3 might be significant to breeding soybeans with lower PPD (Figure 3). According to the study, more than half of the analyzed Chinese landraces possesses the *pdh1* allele (Funatsuki et al., 2014). In this study, the 189 soybean accessions were selected to represent all three ecological regions of soybean cultivation in China and soybeans with different pod dehiscence qualities. However, as more than 20,000 soybean accessions have been preserved (Wang et al., 2006), Hap2 or Hap3 could be used to discover other excellent soybean germplasms with resistance to pod dehiscence. In addition, two typical haplotypes (Hap1 and Hap2) were used to analyze the geographical distribution of Chinese soybean landraces. We found that the favorable haplotype (Hap2) was mostly observed in the HR and SR. Taken together, the above results could facilitate the development of molecular markers for the breeding of soybean accessions with lower PPD.

### Identification of Candidate Genes for Pod Dehiscence in Soybean

Transcription factors play an important role in controlling pod dehiscence. The genetic network directing the morphogenesis of the dehiscence zone in *Arabidopsis* fruit has been identified (Ballester and Ferrandiz, 2017). For example, two members of the MADS-box transcription factor family, SHATTERPROOF1 (SHP1) and SHATTERPROOF2 (SHP2), act redundantly to control silique dehiscence (Liljegren et al., 2000). FRUITFULL (FUL), which also belongs to the MADS-box family, negatively regulates S*HP1/2* expression (Ferrandiz et al., 2000). Moreover, two bHLH transcription factors, INDEHISCENT (IND) and ALCATRAZ (ALC), act downstream of *SHP1/2* (Dong and Wang, 2015). Briefly, the four genes *SHP1*, *SHP2*, *IND*, and *ALC* are expressed at the valve margin to direct dehiscence zone formation. In soybean, the pod-dehiscence-related gene *SHAT1-5* also encodes a NAC transcription factor. Interestingly, previously identified genes that related to pod dehiscence or fruit shedding are homologous to those involved in seed shattering in *Arabidopsis thaliana*. However, Rau et al. (2019) identified a QTL, *qPD5.1-Pv*, represents a novel locus for the shattering trait in common bean (Rau et al., 2019).

In this study, on chromosome 9, two SNPs (AX-94059226 and AX-93762822) were observed under the threshold of FDR < 0.05 in two environments (Env3 and Env5) (Table 2 and Supplementary Table S4). Moreover, we noticed that the SNP AX-93762848 was close to AX-94059226 and AX-93762822, which were observed in one environment with the FDR value < 0.05 (Supplementary Table S4). Based on these SNPs, we identified a candidate gene, *Glyma09g06290*, which is located in the 130kb flanking region of two repeatedly observed SNPs, and the expression of *Glyma09g06290* was significantly increased in later pod growth stages. Furthermore, different expression patterns of *Glyma09g06290* were found in soybean varieties with high and low PPD (Figure 4). Then we sequenced the *Glyma09g06290* gene in a subset of 20 accessions with high PPD and 22 accessions with low PPD. Five SNPs were identified and used to analyze haplotypes, and the haplotype analysis showed that various haplotypes of the putative gene display variation in pod dehiscence (Figure 6). However, the biological functions of this gene should be further investigated. In summary, *Glyma09g06290* might be a putative gene involved in pod dehiscence in soybean.

In addition, the GWAS results revealed that 25 significant SNPs were identified in only one environment except for the SNPs on chromosome 16 (Table 2). However, eleven of them were consecutively distributed on chromosome 8, showed significant marker-trait association, with FDR as low as 3.23 × 10^–03^ (*P*-values = 7.83 × 10^–07^), and were close to *Pod Dehiscence 4-1* (Yamada et al., 2009) (Supplementary Table S4). Interestingly, among these eleven SNPs, seven SNPs were detected within the region of *Glyma08g42110* (one of them was detected within the exon). In addition, the *Glyma08g42110* is homologous to *ATGSL07*, which encodes callose synthase 7 (CalS7), a phloem-specific callose synthase responsible for callose deposition in developing sieve elements during phloem formation. *ATGSL07* mutant plants exhibited moderate reduction in seedling height and produced aberrant pollen grains and short siliques with aborted embryos in *A. thaliana* (Xie et al., 2011). Our further work will also focus on the biological functions of this gene.

## Conclusion

In this study, 163 SNPs were identified using GWAS across multiple environments. In addition, favorable SNPs and six haplotypes relevant to pod dehiscence were identified. Furthermore, based on GWAS results, we identified a candidate gene *Glyma09g06290*. Expression patterns and allelic variation showed that *Glyma09g06290* was associated with the pod dehiscence. Additionally, we also developed a functional marker in *Glyma09g06290* for pod dehiscence in soybean, which may be useful for the breeders. Overall, these results would provide insights for molecular-assisted breeding strategies for resistance to pod dehiscence in soybean.

## Data Availability

Publicly available datasets were analyzed in this study. This data can be found here: http://www.soybase.org/.

## Author Contributions

DY and FH designed the study. DHu, XL, and GK conducted the GWAS. DHu, DHao, WH, ZY, and XH carried out the field experiments. XL, HY, and YL carried out the qRT-PCR analysis. DHu wrote the manuscript. All authors approved the final version of the manuscript.

## Conflict of Interest Statement

The authors declare that the research was conducted in the absence of any commercial or financial relationships that could be construed as a potential conflict of interest.

## Footnotes

1 http://www.soybase.org/soyseq/

2 http://www.plexdb.org

3 http://phytozome.jgi.doe.gov/pz/portal.html

4 http://gsds.cbi.pku.edu.cn/

5 http://www.ncbi.nlm.nih.gov/

6 http://helix.wustl.edu/dcaps/dcaps.html

7 http://www.soybase.org/

## Abbreviations

ANOVA: analysis of variance
BLUP: best linear unbiased prediction
dCAPS: derived cleaved amplified polymorphic sequences
FDR: false discovery rate
GWAS: genome-wide association analysis
*h*^2^: broad-sense heritability
HR: Huang-Huai ecoregion
LD: linkage disequilibrium
MAFs: minor allele frequencies
MAS: marker-assisted selection
NJ: neighbor-joining
NR: northern ecoregion
PPD: the percentage of pod dehiscence
Q–Q plots: quantile–quantile plots
qRT-PCR: quantitative real-time PCR
QTL: quantitative trait locus
RIL: recombinant inbred lines
RNA-Seq: RNA sequencing
SNP: single nucleotide polymorphisms
SR: southern ecoregion.
