# Supplementary material for: Identification of Loci and Candidate Genes Responsible for Pod Dehiscence in Soybean via Genome-Wide Association Analysis Across Multiple Environments
Source: Front Plant Sci. 2019 Jun 21;10:811. doi: 10.3389/fpls.2019.00811 (PMC6598122; doi:10.3389/fpls.2019.00811)
Supplement: Supplementary file 1 [file Data_Sheet_1.pdf]

Identification of Loci and Candidate Genes Responsible for Pod Dehiscence in Soybean via Genome-wide Association Analysis across Multiple Environments

Running title: GWAS for pod dehiscence in soybean

Dezhou Hu[1 †], Guizhen Kan[1 †], Wei Hu[1], Yali Li[1], Derong Hao[2], Xiao Li[1], Hui Yang[1, 3], Zhongyi Yang[1], Xiaohong He[1], Fang Huang[1] * and Deyue Yu[1, 3] *

Correspondence:

Deyue Yu

Email: dyyu@njau.edu.cn

Fang Huang

Email: fhuang@njau.edu.cn

† These authors have contributed equally to this work

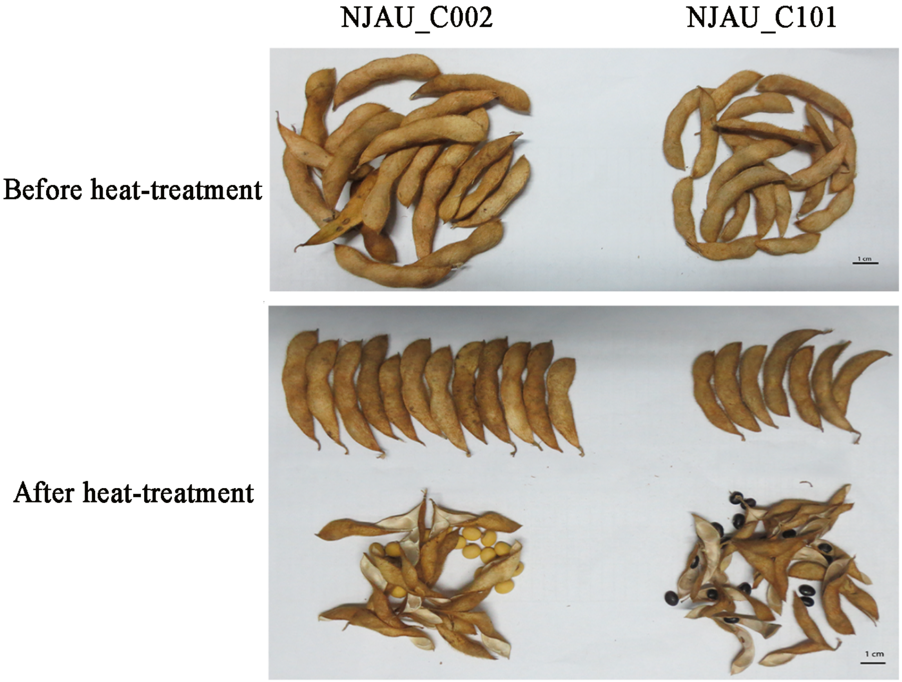

Figure S1. The phenotype of pod dehiscence between two soybean accessions (NJAU_C002 and NJAU_C101) before and after heat-treatment.

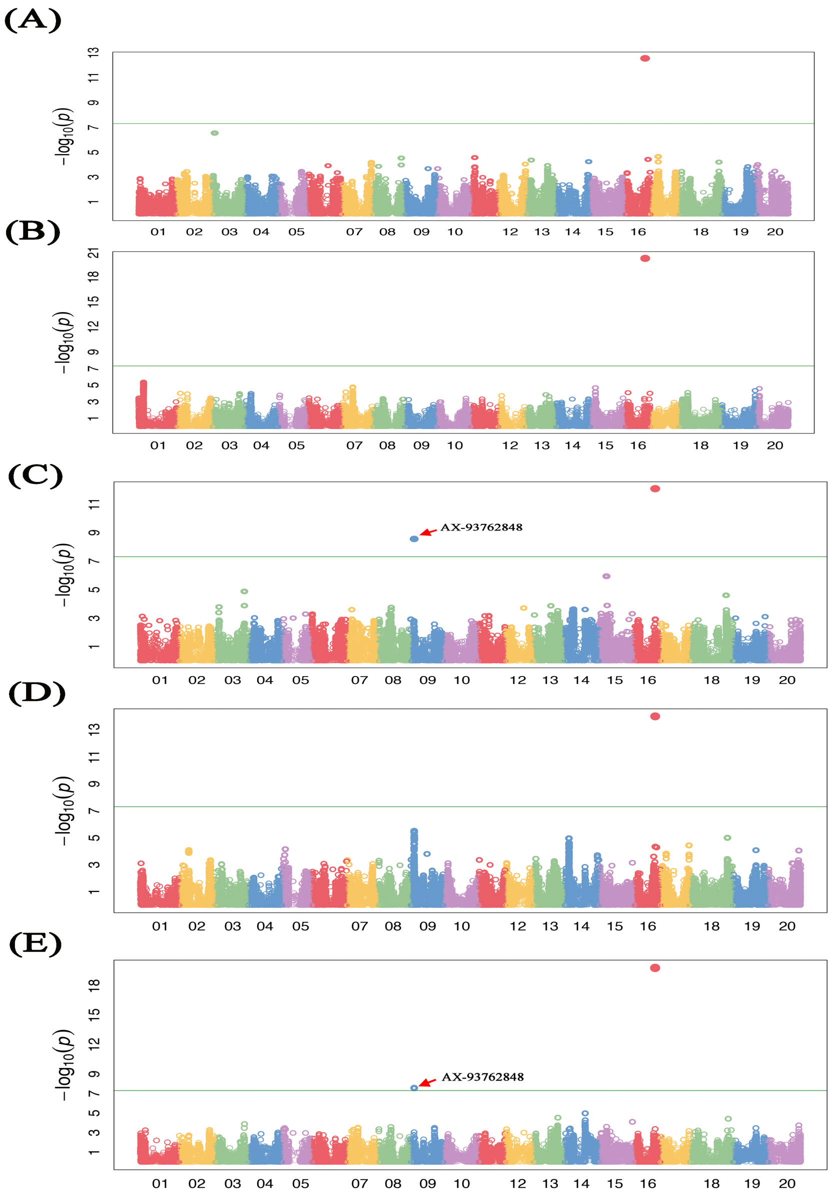

Figure S2. The Manhattan plots for pod dehiscence using MMLM model in the following environments: (A) Env1; (B) Env2; (C) Env3; (D) Env4; (E) Env5. The SNP indicated by the arrow is AX-93762848.

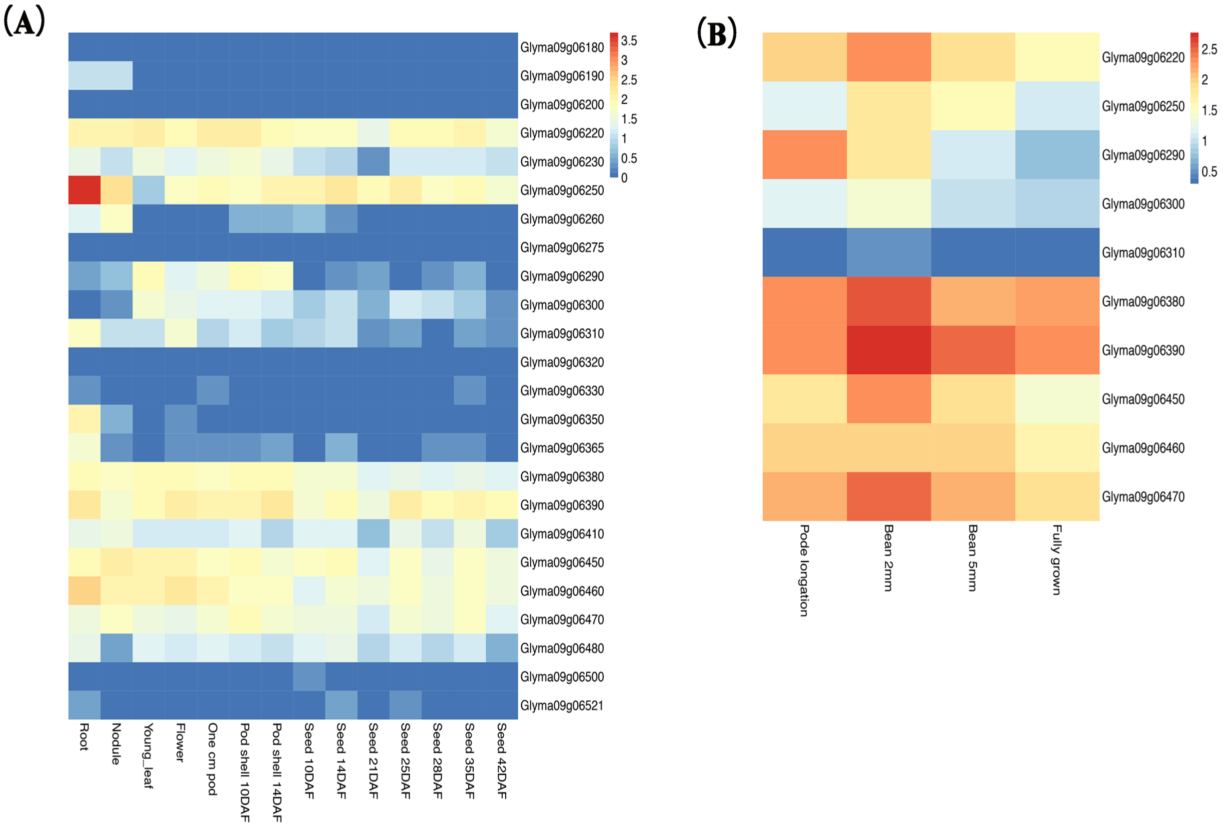

Supplementary Figure S3. Digital expression profiles for 26 genes in various tissues. (A) Expression levels of 26 genes in three vegetative tissues and at different seed developmental stages based on RNA sequencing data. The reads per kilobase million-normalized values were $\log_{10}$–transformed. (B) Expression of 26 genes in four different pod development stages from microarray data. The RMA-normalized values from the microarray data were $\log_{10}$–transformed. The transformed values were employed to create the heat map using R software.

```
                1
Hap1 TGTTGTACGTGCCTGTGCACACTGACTCTCCCTATAATCACTCTCCCACAGAAACTCGTTTTCTAGGCCTGTGGTCTTCTTTTTGTGCGTGTGCGTGCGTCCATACTTGCTTCGCCTAGTAATTATTATAATCCAGTT
Hap2 TGTTGTACGTGGCTGTGCACACTGACTCTCCCTATAATCACTCTCCCACAGAAACTCGTTTTCTAGGCCTGTGGTCTTCTTTTTGTGCGTGTGCGTGCGTCCATACTTGCTTCGCCTAGTAATTATTATAATCCAGTT

     TCCTTATCACTCCAATTATTCAATTACATGGGGCCTCCTCATGTCAAGGAAAAAATTAAAATTTTATTTGCTAGTGAACTTCACTTCCCTATAACTTGAACTATCTTCAACACTAACTTCTCCTCAACCCACACCATT
     TCCTTATCACTCCAATTATTCAATTACATGGGGCCTCCTCATGTCAAGGAAAAAATTAAAATTTTATTTGCTAGTGAACTTCACTTCCCTATAACTTGAACTATCTTCAACACTAACTTCTCCTCAACCCACACCATT

          2                                                                                                       3
     CCCCC-TAAAAGCTTGTTTTTGCCTTCTTCTTTCTCCTCAACATAGCTGACAAAAACAACCACTTCCTCTTTCATTGTTCTCTCCCCCCACTTTCACTATACCCCCCCTTGCTTTACTTTGCATATTTTGCACCCTTT
     CCCCCCTAAAAGCTTGTTTTTGCCTTCTTCTTTCTCCTCAACATAGCTGACAAAAACAACCACTTCCTCTTTCATTGTTCTCTCCCCCCACTTTCACTATACCCCCCATTGCTTTACTTTGCATATTTTGCACCCTTT

                                                                                                      ***
     TAACTTCTATTATCACTCACAATTCTTGTTCTTGGTCTTCTTCCTTTTCTCATCCTTATACACTTCTTTCTTAGATTGTTCTTGTTTGGATTATTTCATGGAGGGTCAACCAGCTAAAAGGAGACGTGTATACTCACT
     TAACTTCTATTATCACTCACAATTCTTGTTCTTGGTCTTCTTCCTTTTCTCATCCTTATACACTTCTTTCTTAGATTGTTCTTGTTTGGATTATTTCATGGAGGGTCAACCAGCTAAAAGGAGACGTGTATACTCACT

       633bp                                                                                                   45
     ------------TTACTATTATGTATGTGTTCCACATATAATATATATTGTCAATTATCAAAGCAACTATATATTTATATAGTATGCTGTACTCTTTTCTTTTTTATTTTAAATATCGTGTATATATTTTTCAAATCTGTT
     ------------TTACTATTATGTATGTGTTCCACATATAATATATATTGTCAATTATCAAAGCAACTATATATTTATATAGTATGCTGTACTCTTTTCTTTTG-ATTTTAAATATCGTGTATATATTTTTCAAATCTGTT
```

Supplementary Figure S4. Comparison of sequence between Hap1 and Hap2. 1-5 represents S_-500, Indel_-230, S_-128, Indel-766 and S-767. *: the translation start codon.**

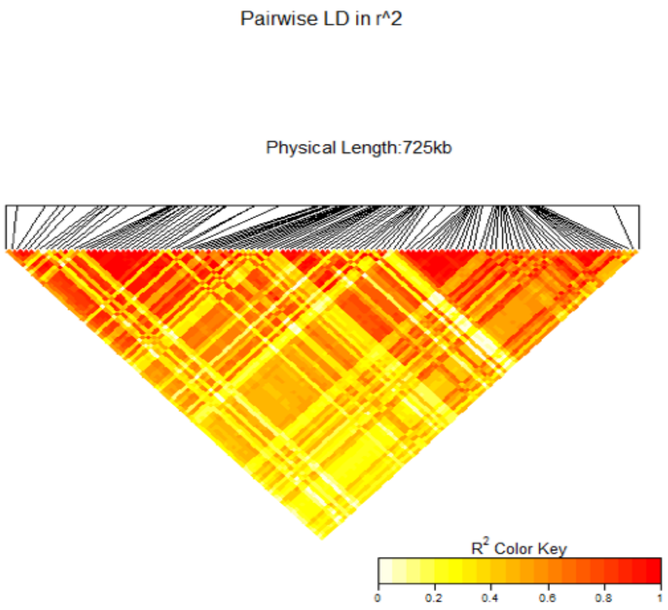

Supplementary Figure S5. LD analysis of the 136 associated SNPs on chromosome 16.

Supplemental Table S2. Primer sequence of qRT-PCR

Gene	Primer	Primer sequence and probe sequence
Glyma09g06290	Forward primer	AGAAGATGATGATGGTGGTGGTGAT
	Reverse primer	TTTGTAGCTCAGTCACCATTTGTTC
Glyma09g06320	Forward primer	GCTCACGAAGGGAGTGAGAT
	Reverse primer	TGACGTGATTCAGAGTACACAC
Glyma09g06390	Forward primer	ATGAAGGCAATGAACCAA
	Reverse primer	TGCGGAATAATGCAACACC
Tubulin	Forward primer	CACTTACGCATCACATAGCA
	Reverse primer	GGAGTTCACAGAGGCAGAG
